# Relationships between serum MCP-1 and subclinical kidney disease: African American-Diabetes Heart Study

**DOI:** 10.1186/1471-2369-13-148

**Published:** 2012-11-14

**Authors:** Mariana Murea, Thomas C Register, Jasmin Divers, Donald W Bowden, J Jeffrey Carr, Caresse R Hightower, Jianzhao Xu, S Carrie Smith, Keith A Hruska, Carl D Langefeld, Barry I Freedman

**Affiliations:** 1 Department of Internal Medicine/Nephrology, Wake Forest School of Medicine, Medical Center Boulevard, Winston-Salem, NC, 27157-1053, USA; 2 Department of Pathology, Wake Forest School of Medicine, Winston-Salem, North Carolina, 27157, USA; 3 Division of Public Health Sciences, Wake Forest School of Medicine, Winston-Salem, North Carolina, 27157, USA; 4 Department of Internal Medicine/Endocrinology/Centers for Diabetes Research and Human Genomics, Wake Forest School of Medicine, Winston-Salem, North Carolina, 27157, USA; 5 Department of Radiology, Wake Forest School of Medicine, Winston-Salem, North Carolina, 27157, USA; 6 Division of Pediatric Nephrology, Washington University School of Medicine, St. Louis, MO, 63110, USA

**Keywords:** African Americans, Albuminuria, Atherosclerotic calcified plaque, Diabetes, GFR, MCP-1

## Abstract

**Background:**

Monocyte chemoattractant protein-1 (MCP-1) plays important roles in kidney disease susceptibility and atherogenesis in experimental models. Relationships between serum MCP-1 concentration and early nephropathy and subclinical cardiovascular disease (CVD) were assessed in African Americans (AAs) with type 2 diabetes (T2D).

**Methods:**

Serum MCP-1 concentration, urine albumin:creatinine ratio (ACR), estimated glomerular filtration rate (eGFR), and atherosclerotic calcified plaque (CP) in the coronary and carotid arteries and infrarenal aorta were measured in 479 unrelated AAs with T2D. Generalized linear models were fitted to test for associations between MCP-1 and urine ACR, eGFR, and CP.

**Results:**

Participants were 57% female, with mean ± SD (median) age 55.6±9.5 (55.0) years, diabetes duration 10.3±8.2 (8.0) years, urine ACR 149.7±566.7 (14.0) mg/g, CKD-EPI eGFR 92.4±23.3 (92.0) ml/min/1.73m^2^, MCP-1 262.9±239.1 (224.4) pg/ml, coronary artery CP 280.1±633.8 (13.5), carotid artery CP 47.1±132.9 (0), and aorta CP 1616.0±2864.0 (319.0). Adjusting for age, sex, smoking, HbA_1c_, BMI, and LDL, serum MCP-1 was positively associated with albuminuria (parameter estimate 0.0021, P=0.04) and negatively associated with eGFR (parameter estimate −0.0003, P=0.001). MCP-1 remained associated with eGFR after adjustment for urine ACR. MCP-1 levels did not correlate with the extent of CP in any vascular bed, HbA_1c_ or diabetes duration, but were positively associated with BMI. No interaction between BMI and MCP-1 was detected on nephropathy outcomes.

**Conclusions:**

Serum MCP-1 levels are associated with eGFR and albuminuria in AAs with T2D. MCP-1 was not associated with subclinical CVD in this population. Inflammation appears to play important roles in development and/or progression of kidney disease in AAs.

## Background

Inflammation, influx of circulating inflammatory cells, synthesis and secretion of chemokines and cytokines play important roles in diabetic kidney disease and atherosclerosis [1,2]. The relationship between serum chemokine monocyte chemoattractant protein-1 (MCP-1, or CCL2) levels with kidney disease and subclinical cardiovascular disease (CVD) has not been evaluated in the African American (AA) population. Macrophages contribute to the pathophysiology of atherosclerosis, albuminuria, diabetic nephropathy (DN), and kidney failure [3,4]. Macrophage trafficking and influx to the blood vessel wall is driven in part by chemokines, and MCP-1 inhibition delays formation of atherosclerotic plaque [5]. In experimental and human DN, macrophages are the principal infiltrating leukocyte population and the degree of macrophage influx and MCP-1 expression in the glomerular and interstitial compartments correlate with albuminuria and kidney function outcome [4,6-8]. Experimentally, MCP-1 suppression ameliorated albuminuria and kidney interstitial disease [7].

Albuminuria and kidney disease are strongly linked with CVD. Presence of a graded association has been demonstrated between estimated glomerular filtration rate (eGFR) and albuminuria, with cardiovascular events, mortality, and presence and severity of coronary artery calcification (CAC) in European-derived populations [9-11]. Despite presence of more severe conventional CVD risk factors, AAs have markedly lower amounts of CAC, carotid artery CP, and aorta CP than EAs [12,13], along with significantly reduced rates of myocardial infarction when provided equal access to healthcare [14-16]. Relationships between conventional CVD risk factors and subclinical CVD do not appear to differ by race, suggesting that novel risk factors including cytokines and genetic variation may contribute to population-specific risks for CP and CVD [17].

As inflammation has emerged at the core pathophysiology of both diabetic nephropathy and atherosclerosis, we sought to investigate the relationships between serum MCP-1 concentrations with albuminuria, kidney function, and vascular calcification in a well-characterized cohort of AAs with type 2 diabetes (T2D) in the African American-Diabetes Heart Study (AA-DHS). Previous reports indicated that inflammation is a protracted process, occurring from the early stages of nephropathy (eGFR >90ml/min/1.73m^2^ and microalbuminuria) in patients with type 1 diabetes (T1D) [18,19]. Presence of inflammation in patients with chronic kidney disease (CKD) has been associated with carotid intimal-medial thickness [20] and increased risk of cardiovascular death [21]. Similarly, vascular endothelial damage begins before it becomes clinically apparent, at early stages of kidney disease (GFR >90 ml/min/1.73m^2^) [22]. Elucidation of inflammatory markers with impact on early kidney disease and vascular dysfunction may guide innovative therapies to prevent or reverse nephropathy and/or vascular damage. We hypothesized that serum MCP-1 concentration, a surrogate of systemic and vascular inflammation, changes in T2D patients in relation to kidney function and vascular integrity. As such, the relationships between serum MCP-1 concentrations with early diabetic nephropathy and vascular calcified plaque were examined.

## Methods

### Study population

The AA-DHS is an observational study conducted on a cohort of self-reported and unrelated AAs with T2D lacking advanced nephropathy. Participants with advanced nephropathy or end-stage renal disease were excluded. Recruitment was conducted from internal medicine clinics and community advertising, as previously published [23]. Briefly, participant examinations were conducted in the Clinical Research Unit of Wake Forest Baptist Medical Center and included interviews for medical history and health behaviors, anthropometric measures, resting blood pressure (BP), electrocardiography, fasting blood sampling (total cholesterol, low density lipoprotein [LDL] cholesterol, high density lipoprotein [HDL] cholesterol, triglycerides, hemoglobin A_1c_ [HbA_1c_, glucose and high sensitivity C-reactive protein [hsCRP]), spot urine collection for albumin:creatinine ratio (ACR), and computed tomography (CT).

History of CVD was provided by participant report and medical record review. Individuals with a history of myocardial infarction or stroke were included; however, CP scores in the coronary arteries were excluded in participants who underwent prior coronary artery bypass grafting and in the carotid arteries in participants who underwent carotid endarterectomy. We assessed eGFR using the simplified MDRD study and CKD-EPI equations [24,25]. Serum creatinine concentration was measured using a modified kinetic Jaffe method and corrected for inter-laboratory differences and calibrated to the Cleveland Clinic [26]. Medications known to influence atherosclerosis (lipid lowering medications) and urine ACR (angiotensin-converting enzyme inhibitors [ACEi] and angiotensin-receptor blockers [ARB]) were recorded. The study was approved by the Institutional Review Board at the Wake Forest School of Medicine and all participants provided written informed consent.

### Vascular imaging

CP in the coronary arteries (CAC), carotid arteries (CarCP), and infrarenal aorta (AorCP) were determined using multidetector computed tomography (MDCT4) with cardiac gating and capable of 500-millisecond temporal resolution using the segmented reconstruction algorithm (LightSpeed Qxi; General Electric Medical Systems, Waukesha, WI, USA). Techniques for the coronary and carotid scans have been described in detail [11]. In brief, participants were placed in the supine position on the CT couch over a quality control calibration phantom (Image Analysis, Inc., Columbia, KY, USA) for scans of the heart and abdomen. The abdomen scan series was used to measure AorCP. Technical factors for this series were: 120 kV, 250 mA, 0.8-second gantry rotation helical mode (7.5 mm/s), 2.5-mm slice thickness, and standard reconstruction kernel. The display field of view was 35 cm, resulting in a pixel dimension of 0.68 by 0.68 mm. CT scans of the three vascular territories were analyzed on a G.E. Advantage Windows Workstation with the SmartScores software package (General Electric Medical Systems) using a modified Agatston scoring method, which adjusts for slice thickness and uses the conventional threshold of 130 Hounsfield units.

### MCP-1 assay

Serum MCP-1 was measured using an enzyme-linked immunosorbent assay (ELISA) (Quantikine® Human CCL2/MCP-1 ELISA; R&D Systems, Minneapolis) in freshly thawed serum samples which had been stored at -80C since collection. Analyses were performed in batches using ELISA kits from a single lot to minimize variability due to manufacturing variation. Intra- and inter-assay coefficients of variation for MCP-1 were 4.0%/3.4% at 62.5 pg/ml and 1.8%/2.1% at 500 pg/ml.

### Statistical methods

Generalized linear models (GLM) were fitted to test for associations between serum MCP-1 concentration and diabetes duration, HbA_1C_, body mass index (BMI), urine ACR, eGFR, CAC, CarCP and AorCP [27]. MCP-1 values greater than 486.7 pg/ml, corresponding to the 95^th^ percentile in the distribution, were winsorized to 486.7 [28]. The Box-Cox method was applied to identify the appropriate transformation best approximating the distributional assumptions of conditional normality and homogeneity of variance of the residuals [29]. This method suggested taking the natural log of (CAC+1), (CarCP+1) and (AorCP+1), (ACR+1), CRP, MDRD and CKD eGFR, the inverse of HbA_1c_ and the inverse square root of BMI to minimize the influence of extremely large covariate values on parameter estimates in the models. No transformation was required for eGFR. GLM models were fitted using the winsorized values of MCP-1 as the dependent variable. After an unadjusted analysis, adjustments for age, sex, smoking, HbA_1c,_ BMI, and LDL levels were incorporated. Urine ACR was analyzed both as categorical variable and as a continuous variable. The models used to test for association between BMI and HbA_1c_ with MCP-1 contained one less variable than the fully adjusted models between MCP-1 and other variables. Inter-active effects between BMI and MCP-1 on kidney function measures were also performed. Interaction effects were evaluated by testing for the direct interaction effect by including the centered product of BMI by MCP-1 and performing the association analysis between MCP-1 and the kidney function measures stratified by BMI where the sample was stratified into two subgroups (non-obese: BMI <30.0 kg/m^2^ and obese: BMI ≥ 30.0 kg/m^2^). Type III sum of squares were also computed to evaluate the effect of eGFR adjusted for ACR (and vice-versa) and all other covariates on the vascular calcification and renal function measures.

## Results

The study included 479 unrelated AAs with T2D (57% women), 50.7% with hypertension (HTN), with mean ± SD (median) age 55.6 ± 9.5 (55.0) years, diabetes duration 10.3 ± 8.2 (8.0) years, and BMI 35.5 ± 8.7 (34.0) kg/m^2^ (Table 1). Participants were stratified by baseline urine ACR into non-albuminuric (urine ACR <30 mg/g; n=300) and albuminuric (urine ACR ≥30 mg/g; n=179). Characteristics of the cohort included serum MCP-1 levels 262.9 ± 239.1 (224.4) pg/ml, hsCRP 1.1 ± 1.8 (0.5) mg/dl, MDRD eGFR 95.2 ± 27.2 (93.3) ml/min/1.73m^2^, CKD-EPI eGFR 92.4 ± 23.3 (92.0) ml/min/1.73m^2^, and urine ACR 149.7 ± 566.7 (14.0) mg/g. There were no between gender differences in serum MCP-1 levels (267.8 ± 242.0 (229.3) pg/ml in women, and 256.5 ± 235.4 (212.8) pg/ml in men, P=0.26). CAC was present in 62.7% of participants, 48.5% had detectable CarCP, and 77.9% detectable AorCP. CKD-EPI and MDRD determined eGFRs were highly correlated (Spearman correlation =0.93).

**Table 1:** Demographic characteristics of study participants by urine albumin: creatinine ratio

**Variable**	**Urine ACR<30 (N=300)**				**Urine ACR≥30 (N=179)**				**ALL (N=479)**				**P-value**
	**Mean**	**Median**	**SD**	**IQR**	**Mean**	**Median**	**SD**	**IQR**	**Mean**	**Median**	**SD**	**IQR**	
Age (years)	55.9	56.0	9.6	13.0	55.0	53.0	9.5	13.0	55.6	55.0	9.5	13.0	0.32
Diabetes duration (years)	9.5	7.0	8.2	8.0	11.6	10.0	8.0	8.0	10.3	8.0	8.2	8.0	0.0007
HTN n (%)	132		(44.0)		111		(62.0)		243		(50.7)		0.0001
BMI (kg/m^2^)	35.5	34.1	8.5	10.0	35.6	33.6	9.2	11.3	35.5	34.0	8.7	10.6	0.83
Systolic BP (mm Hg)	129.9	129.0	16.2	21.0	138.8	137.0	21.6	27.5	133.2	132.0	18.9	24.0	<.0001
Diastolic BP (mm Hg)	76.5	76.0	10.9	14.5	79.4	80.0	11.8	16.0	77.6	77.0	11.3	15.0	0.008
Smoking n (%)													
Past	114		(38.0)		55		(30.7)		169		(35.3)		0.08
Current	61		(20.3)		51		(28.5)		112		(23.4)		
ACEi n (%)	116		(38.7)		75		(41.9)		191		(39.9)		0.48
ARB n (%)	37		(12.3)		34		(19.0)		71		(14.8)		0.04
Lipid lowering med n (%)	148		(51.0)		93		(52.5)		241		(51.6)		0.75
Insulin n (%)	100		(33.3)		93		(52.0)		193		(40.3)		<0.0001
TZD n (%)	37		(12.3)		19		(10.6)		56		(11.7)		0.57

*ACR*, albumin: creatinine ratio; *SD*, standard deviation; *IQR*, interquartile range; *BMI*, body mass index; *BP*, blood pressure; *ACEi*, angiotensin- converting enzyme inhibitor; *ARB*, angiotensin receptor blocker; *HTN*, hypertension; *TZD*, thiazolidinediones.

Subjects with albuminuria had a longer diabetes duration by mean ± SD 2.1 ± 0.2 years (P=0.0007), higher prevalence of HTN (62% vs. 44%, P=0.0001), higher BP values with mean ± SD difference of 8.9 ± 5.4 mmHg in systolic BP (P<0.0001) and 2.9 ± 0.9 mmHg in diastolic BP (P=0.008), and were more often prescribed ARB and insulin (Table 1). Differences in biochemical parameters were also noted, with the albuminuric group having higher HbA1c, total cholesterol, triglycerides, and serum creatinine; and lower fasting glucose and HDL (Table 2). Modeled as a continuous variable, albuminuria was negatively associated with eGFR (parameter estimates and P-values of −0.0014 and 0.04 for CKD-EPI eGFR, and −0.0015 and 0.06 for MDRD eGFR).

**Table 2:** Laboratory and imaging data by urine albumin: creatinine ratio

**Variable**	**Urine ACR<30 (N=296)**				**Urine ACR≥30 (N=175)**				**ALL (N=471)**				**P-value**
	**Mean**	**Median**	**SD**	**IQR**	**Mean**	**Median**	**SD**	**IQR**	**Mean**	**Median**	**SD**	**IQR**	
Fasting Glucose (mg/dl)	151.1	134	66.4	67	140.1	128	55.9	51	169.5	150	77.7	89	<.0001
HbA1c (%)	7.8	7.4	1.7	2.2	8.7	8.4	2.2	2.7	8.1	7.7	2.0	2.4	<.0001
Total Cholesterol (mg/dl)	175.9	169.0	41.0	52.0	189.5	180.0	56.1	62.0	181.0	174.0	47.6	56.0	0.01
HDL-Cholesterol (mg/dl)	49.2	47.0	13.8	16.5	45.3	43.0	13.1	16.0	47.8	46.0	13.6	17.0	0.0008
LDL-Cholesterol (mg/dl)	104.3	98.0	34.3	44.0	112.8	106.0	44.9	58.0	107.4	101.0	38.7	48.0	0.08
Triglycerides (mg/dl)	110.8	95.5	60.2	59.0	166.3	118.0	207.8	102.0	131.5	102.0	138.1	69.0	0.0001
Serum creatinine (mg/dl)	0.9	0.9	0.3	0.3	1.0	1.0	0.3	0.4	1.0	0.9	0.3	0.3	0.01
Serum Calcium	9.6	9.6	0.5	0.5	9.5	9.6	0.4	0.7	9.6	9.6	0.5	0.5	0.33
MDRD eGFR (ml/min)	96.6	93.9	26.3	30.6	92.8	92.3	28.5	38.4	95.2	93.3	27.2	33.6	0.15
CKD-EPI eGFR (ml/min)	93	92.0	21.9	32.4	91.3	92.6	25.5	44.6	92.4	92.0	23.3	35.8	0.53
Urine ACR (mg/g)	8.2	5.3	7.3	8.8	386.9	96.0	878.6	185.0	149.7	14.0	566.7	55.8	NA
hsCRP (mg/dl)	1	0.5	1.4	0.9	1.3	0.6	2.4	1.0	1.1	0.5	1.8	0.9	0.62
MCP-1 (pg/ml)	250.7	214.7	199.5	119.0	283.3	234.1	293.2	135.1	262.9	224.4	239.1	127.4	0.15
CAC (mass score)	222.7	4.5	518.0	170.0	377.8	32.3	784.7	351.8	280.1	13.5	633.8	235.5	0.001
CarCP (mass score)	43.2	0.0	132.8	22.0	53.7	2.3	133.1	44.5	47.1	0.0	132.9	33.0	0.01
AorCP (mass score)	1395	216.5	2426.0	1715.0	1990.0	380.0	3459.0	2301.0	1616.0	319.0	2864.0	1923.0	0.005

*ACR*, albumin: creatinine ratio; *SD*, standard deviation; *IQR*, interquartile range; *HbA1c*, hemoglobin A1c; *HDL*, high density lipoprotein; *LDL*, low density lipoprotein; *eGFR*, estimated glomerular filtration rate; *hsCRP*, high sensitivity C-reactive protein; *MCP-1*, monocyte chemoattractant protein-1; *CAC*, coronary artery calcied plaque; *CarCP*, carotid artery calcified plaque; *AorCP*, infrarenal aorta calcified plaque.

In the univariate analysis, serum MCP-1 levels had negative association with Log (eGFR) and trended towards positive association with Log (urine ACR+1). Adjusted models including demographic characteristics (age, sex, smoking, BMI,) and laboratory values (HbA_1c_, LDL) maintained significant evidence of negative association between MCP-1 and Log (eGFR) (parameter estimate −0.0003, P=0.001) and detected significant positive association with urine ACR after logarithmic transformation (parameter estimate 0.0021, P=0.04) (Table 3). Since urine ACR is associated with eGFR, we analyzed the relationship between MCP-1 and eGFR based on adjusting for Log (ACR+1), in a fully adjusted model. Compared to ACR alone (parameter estimate −0.0135, P=0.05), MCP-1 had the strongest association with CKD-EPI eGFR (parameter estimate −0.0004, P=0.002) (Table 4).

**Table 3:** MCP-1 associations in the unadjusted and fully adjusted models

**Outcome**	**Unadjusted**			**Adjusted**		
	**Estimate**	**StdErr**	**P-value**	**Estimate**	**StdErr**	**P-value**
Log(Diabetes duration)	0.00060	0.00033	0.07	0.0003	0.0003	0.34
1/(HbA1c)	2.71E-06	1.248E-05	0.83	−7.70E-06	1.23E-05	0.53
1/sqrt(BMI)	−1.37E-05	8.53E-06	0.11	−2.02E-05	8.28E-06	0.01
Log(hsCRP)	0.0003	0.0006	0.54	−0.0001	0.0007	0.87
Log(CAC+1)	0.0006	0.001	0.57	−0.0003	0.0015	0.82
Log(CarCP+1)	0.0001	0.0008	0.86	−0.0012	0.0011	0.31
Log(AorCP+1)	0.0013	0.0012	0.25	−0.001	0.0015	0.48
Log(Urine ACR+1)	0.0013	0.0007	0.09	0.0021	0.001	0.04
Log(MDRD eGFR)	−0.0005	0.0001	<.0001	−0.0005	0.0001	0.006
Log(CKD-EPI eGFR)	−0.0004	0.0001	<.0001	−0.0003	0.0001	0.001

*MCP-1*, monocyte chemoattractant protein-1; *HbA1c*, hemoglobin A1c; *BMI*, body mass index; *hsCRP*, high sensitivity C-reactive protein; *CAC*, coronary artery calcified plaque; *CarCP*, carotid artery calcified plaque; *AorCP*, infrarenal aorta calcified plaque; *ACR*, albumin: creatinine ratio; *eGFR*, estimated glomerular filtration rate.

Adjusted model includes age, sex, smoking, HbA1c, BMI, and LDL-cholesterol.

**Table 4:** Association between MCP-1, eGFR, and ACR in the fully adjusted model

**Dependent**	**Parameter**	**Estimate**	**StdErr**	**P-value**
Log(MDRD eGFR)	MCP-1	-0.0004	0.0001	0.002
Log(MDRD eGFR)	Log(ACR+1)	-0.0133	0.0078	0.08
Log(CKD-EPI eGFR)	MCP-1	-0.0004	0.0001	0.002
Log(CKD-EPI eGFR)	Log(ACR+1)	-0.0135	0.0072	0.05

*MCP-1*, monocyte chemoattractant protein-1; *eGFR*, estimated glomerular filtration rate; *ACR*, albumin: creatinine ratio.

Covariates included in the model are age, sex, smoking, HbA1c, BMI, and LDL.

We next assessed whether there is a correspondence between MCP-1, ACR, eGFR, and vascular CP. No association was detected between MCP-1 and CAC, CarCP, or AorCP in either unadjusted or adjusted models (Table 3). However, albuminuria was independently and significantly associated with vascular CP in all three vascular beds, while eGFR did not exhibit an association (Table 5).

**Table 5:** Type III mean squares and association between CP, ACR, and MCP-1

**Dependent**	**Model**	**Predictor**	**Estimate**	**StdErr**	**P-value**
CAC	ACR + all covariates in the adjusted model	Log(ACR+1)	1.5E-01	6.6E-02	0.02
	eGFR + all covariates in the adjusted model	eGFR	1.7E-04	4.3E-03	0.96
	ACR and MCP-1 + all covariates in the adjusted model	Log(ACR+1)	1.5E-01	6.6E-02	0.02
		MCP-1	-1.7E-04	1.1E-03	0.87
	eGFR and MCP-1+ all covariates in the adjusted model	eGFR	2.0E-04	4.4E-03	0.96
		MCP-1	6.0E-05	1.1E-03	0.95
	ACR, eGFR and MCP-1+ all covariates in the adjusted model	eGFR	6.9E-04	4.4E-03	0.87
		Log(ACR+1)	1.5E-01	6.6E-02	0.02
		MCP-1	-1.4E-04	1.1E-03	0.89
CarCP	ACR + all covariates in the adjusted model	Log(ACR+1)	1.1E-01	5.1E-02	0.02
	eGFR + all covariates in the adjusted model	eGFR	-4.3E-03	3.4E-03	0.20
	ACR and MCP-1 + all covariates in the adjusted model	Log(ACR+1)	1.1E-01	5.2E-02	0.02
		MCP-1	3.3E-05	8.5E-04	0.96
	eGFR and MCP-1+ all covariates in the adjusted model	eGFR	-4.2E-03	3.4E-03	0.21
		MCP-1	5.7E-05	8.6E-04	0.94
	ACR, eGFR and MCP-1+ all covariates in the adjusted model	eGFR	-3.9E-03	3.4E-03	0.25
		Log(ACR+1)	1.1E-01	5.2E-02	0.03
		MCP-1	-1.1E-04	8.6E-04	0.90
AorCP	ACR + all covariates in the adjusted model	Log(ACR+1)	1.9E-01	6.9E-02	0.005
	eGFR + all covariates in the adjusted model	eGFR	-5.2E-03	4.4E-03	0.24
	ACR and MCP-1 + all covariates in the adjusted model	Log(ACR+1)	1.9E-01	6.9E-02	0.006
		MCP-1	6.4E-04	1.1E-03	0.56
	eGFR and MCP-1+ all covariates in the adjusted model	eGFR	-4.8E-03	4.5E-03	0.28
		MCP-1	7.7E-04	1.1E-03	0.49
	ACR, eGFR and MCP-1+ all covariates in the adjusted model	eGFR	-4.2E-03	4.5E-03	0.34
		Log(ACR+1)	1.9E-01	6.9E-02	0.007
		MCP-1	4.9E-04	1.1E-03	0.66

*CP*, calcified plaque; *ACR*, albumin: creatinine ratio; *eGFR*, estimated glomerular filtration rate; *MCP-1*, monocyte chemoattractant protein-1; *CAC*, coronary artery calcified plaque; *CarCP*, carotid artery calcified plaque; *AorCP*, infrarenal aorta calcified plaque.

The covariates included in the adjusted model are age, sex, smoking, HbA1c, BMI, and LDL.

Relationships between serum MCP-1 with diabetes duration, BMI, and hsCRP were also assessed. No correlations were observed between serum MCP-1 and diabetes duration or hsCRP. Serum MCP-1 levels correlated with BMI, and this remained significant in the adjusted model (P=0.01) (Table 3). To assess whether BMI impacts the relationship between MCP-1 and kidney function, association analyses were run with participants stratified as obese (BMI ≥30.0) and non-obese (BMI <30.0). We found no evidence of an interaction effect between BMI and MCP-1 on either eGFR or urine ACR. As shown in Table 6, MCP-1 association parameters in obese participants were similar to those in the non-obese group (−0.0004 vs. -0.0004, P=0.75, for the interaction effect on Log (eGFR); and 0.0017 vs. 0.0013, P=0.68, for the effect on Log (urine ACR+1)). Evidence of an association between MCP-1 and kidney function remained significant in BMI stratified analyses, with meta-analysis P-value = 0.001 for Log (eGFR) and 0.04 for Log (urine ACR+1).

**Table 6:** Fully adjusted MCP-1 associations stratified by BMI

	**CKD-EPI eGFR**					**Urine ACR**			
	**N**	**Estimate**	**StdErr**	**P-meta**	**P-inter**	**Estimate**	**StdErr**	**P-meta**	**P-inter**
BMI < 30.0	134	-0.0004	0.0002	0.001	0.75	0.0017	0.0015	0.04	0.68
BMI ≥ 30.0	345	-0.0004	0.0001			0.0013	0.0009		

*eGFR*, estimated glomerular filtration rate; *ACR*, albumin: creatinine ratio; *BMI*, body mass index.

Results were adjusted for age, sex, smoking, HbA1c, and LDL-cholesterol.

eGFR and ACR values were logarithmically transformed.

P-meta is the P-value observed between MCP-1 and each outcome stratified by BMI.

P-inter is the interaction P-value for the association difference between MCP-1 and each outcome in each BMI subgroup.

## Discussion and conclusion

This large cross-sectional study characterized relationships between serum MCP-1, albuminuria, eGFR and CP in the understudied AA population with T2D. After adjusting for covariates, higher serum MCP-1 levels associated positively with albuminuria and negatively with eGFR. In contrast, serum MCP-1 did not independently associate with atherosclerosis and subclinical CVD measured as CP, suggesting differential molecular relationships between inflammation, risk for kidney disease, and CVD in AAs with T2D.

The pathophysiologic connection between atherosclerosis, CAC, albuminuria and kidney dysfunction is poorly understood at the molecular level. Previous studies demonstrated that MCP-1 is involved in the pathophysiology of atherosclerosis and DN in T1D and T2D [5,7]. MCP-1 is synthesized and secreted by a myriad of cells (monocytes, macrophages, endothelial cells, renal mesangial and tubular cells); and both tissue and systemic cells can contribute to detectable serum MCP-1 levels. In the hyperglycemic milieu, MCP-1 is produced by resident renal endothelial cells, mesangial cells, podocytes, and tubular epithelial cells; as well as by circulating or infiltrating monocytes/macrophages [30]. Several reports attest to the positive correlation between *tissue* MCP-1 expression and *urine* levels with albuminuria, mesangial proliferation, and interstitial fibrosis in a wide range of kidney diseases in humans [8,31-36]. In small studies comprised of European-derived participants with T1D or T2D, ELISA-based measurements of *serum* MCP-1 did not correlate with albuminuria [30,34]. It has been proposed that while the histopathology in diabetic kidney disease has remarkable similarity between type 1 and type 2 diabetes, and between population groups, the pathogenetic background may differ between AAs and EAs, and T2D or T1D [37]. Other longitudinal studies comprised of EAs with T1D, found that *urine* MCP-1 levels were significantly higher in patients with early nephropathy (GFR<90 ml/min and microalbuminuria) relative to those without nephropathy, with no difference in *serum* MCP-1 levels. [18,19] Relative to EAs, it is possible that inflammatory pathways are upregulated in AAs. Previous studies have shown that AAs have higher serum CRP and interleukin-6 (IL-6) concentrations and display heightened oxidative stress and inflammation based on *in vitro* human umbilical vein endothelial cells (HUVECs) studies [38,39]. It is biologically plausible that MCP-1 may play differential roles in the pathophysiology of DN based on the type of diabetes and ethnic background.

We originally postulated that inflammation is a common mediator for both subclinical kidney disease and CVD in AAs with T2D and that systemic MCP-1 levels would correlate with markers of kidney disease and atherosclerosis. We found that a higher burden of vascular calcification was present in those with albuminuria, but CP did not associate with serum MCP-1 levels. Other studies demonstrated that *serum* MCP-1 levels correlate with CVD outcomes following acute coronary events, independent of traditional CVD risk factors [40]. Nevertheless, these studies did not examine the effect of serum MCP-1 on CV events based on kidney function or independent of the association with urine albumin excretion and eGFR. As in the present report, a large population-based sample from the Dallas Heart Study did not observe an association between serum MCP-1 and CAC after adjusting for age and other covariates [41].

This is the first report of which we are aware detecting associations between serum MCP-1 with albuminuria and eGFR in AA patients with T2D and early nephropathy. Study participants were AAs without advanced kidney disease and no differences in serum MCP-1 levels were seen across genders. The nature of the factors determining elevated concentrations of serum MCP-1 in patients with T2D and early DN remains unknown. It is possible that high MCP-1 expression in the interstitial kidney macrophages leads to elevated systemic levels of MCP-1 proportional to the inflammatory and nephropathy stage. Another possibility, not mutually exclusive, is that serum MCP-1 levels are elevated in patients with early nephropathy due to dysregulated activation of systemic leukocytes. Indeed, several studies confirm an aberrant production of inflammatory cytokines and chemokines by circulating lymphocytes and monocytes in T2D patients with nephropathy [42]. Decreased filtration of extra-renally synthesized MCP-1 is less likely, since a minority of participants had an eGFR below 60 ml/min/1.73m^2^.

In addition to roles of MCP-1 in atherosclerosis and kidney disease, several studies implicated MCP-1 in the pathophysiology of obesity and insulin resistance [43,44]. In our sample of AAs with T2D, significant correlations were observed between MCP-1 and BMI, but not with diabetes duration or HbA_1c_. The association between adipose tissue and MCP-1 raised the question whether the link between serum MCP-1 and renal function parameters could have been driven by the high prevalence of obesity in this cohort. Adjustment for BMI and cholesterol failed to modify the association and BMI-stratified effect sizes were not statistically different between obese and non-obese strata. As such, relationships between serum MCP-1 and kidney function were not impacted by obesity.

Significant relationships between MCP-1 with eGFR and albuminuria, coupled with lack of association with CP, imply that MCP-1 does not mediate joint pathways implicated in co-existing kidney and CVD. However, the lack of a cross-sectional association between MCP-1 and burden of CP in AAs does not exclude a role for this molecule in the inflammatory component of atherosclerosis. Previous studies have shown that serum MCP-1 levels are higher in patients with active angina (compared to those with stable coronary disease), and higher levels predicted future coronary events and mortality following an acute coronary event [45,46]. In addition, serum MCP-1 levels have been associated with immunohistochemical indices of inflammation and matrix remodeling in the coronary atherosclerotic plaques of non-human primates [47]. The role of MCP-1 in CP should also be explored in EAs, a population with higher burden of vascular calcification than AAs [12,13].

This study has important strengths and some limitations. AAs are known to display different patterns of nephropathy and CVD morbidity relative to EAs. The large and well phenotyped AA sample enabled simultaneous evaluation of a molecular biomarker potentially impacting albuminuria, eGFR, and subclinical atherosclerosis. Preserved kidney function in AA-DHS participants lessens concern that altered serum MCP-1 levels were due to kidney failure, whether mediated by poor renal excretion or inflammation-driven overproduction. Limitations include the cross-sectional nature of study measurements, rendering inability to secure a causal relationship between MCP-1 and early DN. Longitudinal studies characterizing relationships between MCP-1 and albuminuria and eGFR are warranted and could provide support for pharmacological MCP-1 inhibition during the incipient stages of DN [48]. Recent studies in mouse models suggest such treatment has promise [49].

In conclusion, MCP-1 serum concentrations manifest positive association with albuminuria and negative association with eGFR in AAs with T2D; without association with subclinical atherosclerosis. Relationships between MCP-1, albuminuria, eGFR, and vascular CP need to be evaluated in EAs and non-diabetic AAs. MCP-1 inhibition could provide a novel therapeutic strategy to prevent diabetic kidney disease in AAs with T2D.

## Abbreviations

HbA_1c_: Hemoglobin A1c; AA(s): African American(s); AA-DHS: African American-Diabetes Heart Study; ACEi: Angiotensin-converting enzyme inhibitor; ACR: Urine albumin: creatinine ratio; AorCP: Infrarenal aorta calcified plaque; ARB: Angiotensin receptor blocker; BMI: Body mass index; BP: Blood pressure; CAC: Coronary artery calcified plaque; CarCP: Carotid artery calcified plaque; CCL2: Chemokine (C-C motif) ligand 2; CKD: Chronic kidney disease; CKD-EPI: Chronic Kidney Disease Epidemiology; CP: Calcified plaque; CT: Computed tomography; CVD: Cardiovascular disease; DN: Diabetic nephropathy; EA(s): European American(s); eGFR: Estimated glomerular filtration rate; HDL: High density lipoprotein; hsCRP: High sensitivity C-reactive protein; HTN: Hypertension; LDL: Low density lipoprotein; MCP-1: Monocyte chemoattractant protein-1; MDRD: Modification of Diet in Renal Disease Study; T1D: Type 1 diabetes; T2D: Type 2 diabetes; TZD: Thiazolidinediones.

## Competing interests

The authors report no conflicts of interest.

## Authors’ contributions

MM – study design, manuscript preparation; TCR – study design, MCP-1 assays, manuscript preparation; JD – statistical analysis, manuscript editing; DWB – manuscript editing; JJC – radiologic imaging and interpretation, manuscript editing; CRH – radiologic imaging interpretation; JX – database management; CSS – participant recruitment, manuscript editing; CDL – statistical analysis, manuscript editing; KAH – study design, manuscript editing; BIF – study design, participant recruitment, supervision of data analyses, manuscript preparation. All authors read and approved the final manuscript.

## Pre-publication history

The pre-publication history for this paper can be accessed here:

http://www.biomedcentral.com/1471-2369/13/148/prepub
